# Using machine learning with intensive longitudinal data to predict depression and suicidal ideation among medical interns over time

**DOI:** 10.1017/S0033291722003014

**Published:** 2023-09

**Authors:** Adam G. Horwitz, Shane D. Kentopp, Jennifer Cleary, Katherine Ross, Zhenke Wu, Srijan Sen, Ewa K. Czyz

**Affiliations:** 1 Department of Psychiatry, University of Michigan, Ann Arbor, MI, USA; 2 Department of Psychology, University of Michigan, Ann Arbor, MI, USA; 3 Department of Biostatistics, University of Michigan, Ann Arbor, MI, USA

**Keywords:** Daily diary, depression, intensive longitudinal data, machine learning, medical interns, mood, passive sensing, suicidal ideation

## Abstract

**Background:**

Use of intensive longitudinal methods (e.g. ecological momentary assessment, passive sensing) and machine learning (ML) models to predict risk for depression and suicide has increased in recent years. However, these studies often vary considerably in length, ML methods used, and sources of data. The present study examined predictive accuracy for depression and suicidal ideation (SI) as a function of time, comparing different combinations of ML methods and data sources.

**Methods:**

Participants were 2459 first-year training physicians (55.1% female; 52.5% White) who were provided with Fitbit wearable devices and assessed daily for mood. Linear [elastic net regression (ENR)] and non-linear (random forest) ML algorithms were used to predict depression and SI at the first-quarter follow-up assessment, using two sets of variables (daily mood features only, daily mood features + passive-sensing features). To assess accuracy over time, models were estimated iteratively for each of the first 92 days of internship, using data available up to that point in time.

**Results:**

ENRs using only the daily mood features generally had the best accuracy for predicting mental health outcomes, and predictive accuracy within 1 standard error of the full 92 day models was attained by weeks 7–8. Depression at 92 days could be predicted accurately (area under the curve >0.70) after only 14 days of data collection.

**Conclusions:**

Simpler ML methods may outperform more complex methods until passive-sensing features become better specified. For intensive longitudinal studies, there may be limited predictive value in collecting data for more than 2 months.

Intensive longitudinal methods (ILMs), including self-report from ecological momentary assessments (EMAs) or daily diaries, and passive sensing from smartphones and smartwatches, have grown increasingly popular for the detection and prediction of psychological constructs (e.g. Hamaker & Wichers, 2017). These approaches provide methodological advantages (e.g. reduction in recall bias, capturing short-term variability) and offer significant opportunities for detecting and responding to risk states sooner or more proximally, particularly among vulnerable populations (Bolger & Laurenceau, 2013). Studies have demonstrated the ability of ILMs to improve the prediction of near-term mental health outcomes, such as depression (e.g. Pedrelli et al., 2020; Yim et al., 2020) and suicidal ideation (SI; e.g. Ammerman & Law, 2022; Rabasco & Sheehan, 2022). However, most of these studies contain relatively small and primarily clinical samples, and have high-burden participation designs (e.g. responding to surveys multiple times per day) that may not generalize to non-research settings. Further, ILM studies examining depression and SI have varied significantly in length of the study period (e.g. Colombo et al., 2019; Rabasco & Sheehan, 2022), ranging from several days to several months. Adherence to assessments tends to decay over time (e.g. Czyz, King, & Nahum-Shani, 2018; Glenn et al., 2020), with and drop-out being more likely among participants with greater clinical severity of symptoms (e.g. Colombo et al., 2019; Gershon, Kaufmann, Torous, Depp, & Ketter, 2019). There is a need to examine the utility of ILMs in detecting periods of heightened risk or decline in functioning in the context of real-world, naturalistic settings, and with particular focus on optimizing the duration of data collection such that improved detection is weighted against the risks of overburdening participants.

With the volume of EMA and passive-sensing data readily available through mobile applications and wearable sensors, researchers have increasingly turned to machine learning (ML) methods to process large datasets (Torous et al., 2018). With respect to mental health research, recent studies have highlighted the potential for ML techniques to form more complex models that improve the prediction of depression and suicide-related outcomes (e.g. Burke, Ammerman, & Jacobucci, 2019; Ribeiro, Huang, Fox, Walsh, & Linthicum, 2019; Yim et al., 2020). Despite advantages, complex and thus less interpretable (‘black box’) ML models have also been criticized for limited clinical utility or generalizability, particularly when devoid of theory (e.g. Cox, Moscardini, Cohen, & Tucker, 2020), and complex ML models have not been consistently associated with better prediction than traditional methods (e.g. Jacobucci, Littlefield, Millner, Kleiman, & Steinley, 2021; Littlefield et al., 2021). Additional research is needed to examine how simpler ML approaches perform relative to more complex ML approaches in intensive longitudinal studies, and how performance is impacted by the inclusion of passive-sensing data.

To test simpler *v.* more complex ML approaches in predicting mental health outcomes, we leveraged a sample of first-year physicians participating in the Intern Health Study (Fang et al., 2022). Depression is particularly common among training physicians during medical internship, with 25–30% of trainees screening positive for depression (Mata et al., 2015), which presents an increased risk for medical errors (Fahrenkopf et al., 2008) and for these medical professionals leaving the field (Williams et al., 2010). SI also increases during medical internship and physicians are more likely than the general population to die by suicide (Goldman, Shah, & Bernstein, 2015). A recent investigation demonstrated that intensive longitudinal data (ILD) collected each day over a 60 day period during the first quarter of medical internship improves the prediction of eventual depression and SI at the end of the quarter, over and above initial symptom- and trait-level indicators (Horwitz et al., 2022). Yet, data collection past a certain point in time may be providing only limited additional predictive utility, while potentially contributing to respondent burden and drop out. The present study seeks to illuminate this tradeoff by utilizing ILD from a large sample of training physicians to examine their predictive accuracy for identifying depression and SI over time. Further, despite significant excitement around ML models, findings have been somewhat mixed with respect to their advantage in predicting clinical outcomes, and there is need for additional research comparing simpler (e.g. linear models, self-report data only) *v.* more complex (e.g. non-linear models, incorporating passive-sensing data) ML models.

Our primary study questions are as follows:

1. During the first quarter (i.e. 92 days) of internship, how soon can intensive longitudinal data (ILD) predict end-of-quarter outcomes of depression and suicidal ideation with similar accuracy (e.g. within 1 standard error of the area under the curve (AUC)) compared to the ‘full’ model using all 92 days of observations?
  - In what ways do facets of model complexity, such as non-linearity and use of passive-sensing features influence outcomes? Specifically, how do linear elastic net regressions (ENR) compare to non-linear random forest (RF) models, and does the inclusion of passive-sensing variables improve model performance over and above self-reported daily mood features?
  - How does engagement with daily self-report items (adherence rate) impact the timeline for reaching these accuracy thresholds (e.g. is accuracy improved sooner for individuals with higher, *v.* lower, adherence)?

## Methods

### Participants

The Intern Health Study is an ongoing, prospective cohort study of first-year medical residents at medical residency institutions in the USA (Sen et al., 2010). Participants included in this analysis were 2459 first-year training physicians from over 300 residency institutions across the USA who enrolled in the study during the 2020–2021 academic year. The sample had 55.1% female, and the mean age was 27.6 years (s.d. = 2.7). Racial/ethnic distribution was as follows: 52.5% White, 24.5% Asian, 5.4% Black, 5.0% Hispanic/Latinx, 9.4% multi-racial, and 0.4% other race.

### Measures

#### Depression and suicidal ideation

The Patient Health Questionnaire-9 (PHQ-9; Kroenke, Spitzer, & Williams, 2001) assesses the nine DSM-5 depressive symptoms, with each item rated on a 4-point Likert scale (0–27 full scale range) for frequency of being bothered by a particular symptom in the past 2 weeks, ranging from ‘not at all’ to ‘nearly every day’. A cut-point of 10 was used to indicate the presence of depression at the follow-up survey (Manea, Gilbody, & McMillan, 2015); this threshold was selected as it represents at least moderate depression. The final item from the PHQ-9 was used to assess frequency of SI, ‘thoughts that you would be better off dead or hurting yourself in some way’. This item was dichotomized based on the presence (score of 1 or higher) or absence (score of 0) of any SI; thus, score of 1 represents the presence of thoughts of suicide occurring at least several days. At the follow-up survey, 18.5% of interns had scores above 10 on the PHQ-9 (an increase from 7.9% prior to internship) and 6.8% indicated the presence of SI (an increase from 3.6% prior to internship).

#### Daily mood diary

Daily mood was assessed with a single item using a mobile application, ‘On a scale of 1 (lowest) to 10 (highest), how was your mood today?’ Features derived from these assessments during the first quarter included mean scores, variability in scores (standard deviation), and percent of assessments completed (missingness).

#### Fitbit wearable measures

Sleep and activity features were derived from wearable Fitbit Charge 4 devices, with mean, variability, and missingness of the following daily-level variables: total sleep, sleep start time, sleep end time, sleep efficiency (percentage of time in bed relative to time asleep), total steps, active minutes (sedentary, lightly active, fairly active, very active), and resting heart rate. Previous studies have demonstrated the reliability and validity of using data collected by Fitbits to measure these constructs (for a review, see Evenson, Goto, & Furberg, 2015).

### Procedures

Graduating medical students who were matched to participating residency institutions were invited to take part in the study 3 months prior to the start of internship. Prior to internship start, participants provided informed consent online and downloaded the study mobile application. Participants were provided with a Fitbit to provide continuous wearable sensor data. Daily mood scores were collected through the study's mobile application and participants were prompted to complete this measure between 5pm and 10pm. The PHQ-9 assessment was also completed through the study app at the end of the first quarter. The study was approved by the institutional review board at the University of Michigan.

### Data analytic plan

#### Data preparation

To investigate the study hypotheses, we fit a series of ML models using daily assessments of self-report and passively-collected Fitbit variables that were aggregated within persons for a given number of days. This process was repeated for each day during the study period and performance metrics were estimated using nested cross validation. Performance was compared across days, across different sets of predictor variables, and across groups defined by rates of missing daily mood observations. All data preprocessing and ML models were implemented using R statistical software (R Core Team, 2021) with the *tidymodels* library (Kuhn & Wickham, 2020). Data were originally collected in long format with 92 rows per person (one observation of each variable per day for 92 days). We iteratively calculated three statistics (mean, standard deviation, and percent missing) for the daily mood and 10 Fitbit variables (33 total features) by summarizing daily observations from day 1 to day *t*. When daily observations were missing, but the minimum number need to calculate summary statistics was present (at least two observations for means and three for standard deviations), statistics were calculated using the available observations and missing values were omitted.

Using all available data, we predicted two outcomes: presence of SI and presence of depression at the end of the first quarter of internship. We utilized one linear (ENR – a penalized version of a generalized linear model, with a logit link function) and one non-linear (RF) ML algorithm. Two sets of predictor variables were supplied to the ML algorithms: summaries of daily mood ratings and a combination of daily mood summaries and Fitbit summaries. In instances where there were not enough daily observations to calculate a summary statistic, the median value of that statistic from the training data was assigned. The resulting eight unique models (two outcomes, two ML algorithms, two sets of predictors) were estimated iteratively to simulate predicting end-of-quarter outcomes on day *t*, using the data available up to that point in time.

#### Model performance and validation

ML model performance was assessed using nested cross validation. Nested cross validation is useful for reducing bias when making comparisons across several models. This scheme uses a nested loop structure to validate models in two steps (for a detailed explanation, see Varma & Simon, 2006). Within the inner loop, three-fold cross validation was used to tune model hyperparameters. For ENR a 3 × 3 gird search was performed to select the optimal combination of values for the *α* (penalty) and *λ* (mixture) hyperparameters (Friedman, Hastie, & Tibshirani, 2010). For RFs, the number of trees was set to 100 and a 3 × 3 grid search was performed to select the number of variables used to split at each node and the minimum number of observations required for a node to be split further (Wright & Ziegler, 2017). Splits in the RF models were guided by the Gini impurity index. Across both algorithms, default grid search values from the *tidymodels* library were used. The combination of hyperparameters from the inner loop that produced the greatest AUC was selected. Within the outer loop, repeated *k*-fold cross validation (repeats = 3, *k* = 5) was used to estimate out-of-sample performance. During each iteration, the model returned by the inner cross-validation loop was used to generate predicted class probabilities for the test fold of the outer loop. From these probabilities, AUC was calculated. This process resulted in a set of 15 estimates of the AUC per day. For each day, the AUC ± 1 standard error was reported. In the calculation of the standard error of repeated *k*-fold cross-validation results, some sources (e.g. Kuhn & Johnson, 2013) use square root of the number of repeats multiplied by *k* (i.e. 5 × 3) in the denominator term. This can result in optimistically biased standard errors. Here, we use a more conservative denominator term: the square root of *k* (i.e. 5).

Comparing cross-validation results across models is complex and remains an open question in the ML literature (Bates, Hastie, & Tibshirani, 2021). This issue is further complicated when comparing autocorrelated results across days, as in the current study. To the best of our knowledge, no formal means of comparison has been established for this scenario. Instead, we rely on a commonly used the heuristic one-standard-error rule (Kuhn & Johnson, 2013) to identify the day upon which a model trained on the data available to date approximated the predictive performance of a model trained on data available on the final day of the study period.

To investigate the influence of rates of missing daily mood observations, data were split into separate groups based on missingness and a series of ML models were trained within each subsample. The best performing model architecture from above (ENR with mood-only predictors, including missingness) was utilized for these analyses. For models predicting moderate depression, participants were split into groups with high (>67%), medium (between 33% and 67%), or low (<33%) proportions of missing daily mood observations within each daily dataset. Since these rates were recalculated each day, single individuals could be in different missingness groups at different points of the quarter. Given the power constraints associated with low prevalence of SI, participants were assigned to either a high (⩾50% of observations) or low (<50% of observations) missingness group within each daily dataset.

## Results

### Predicting mental health outcomes over time

**Fig. 1.**
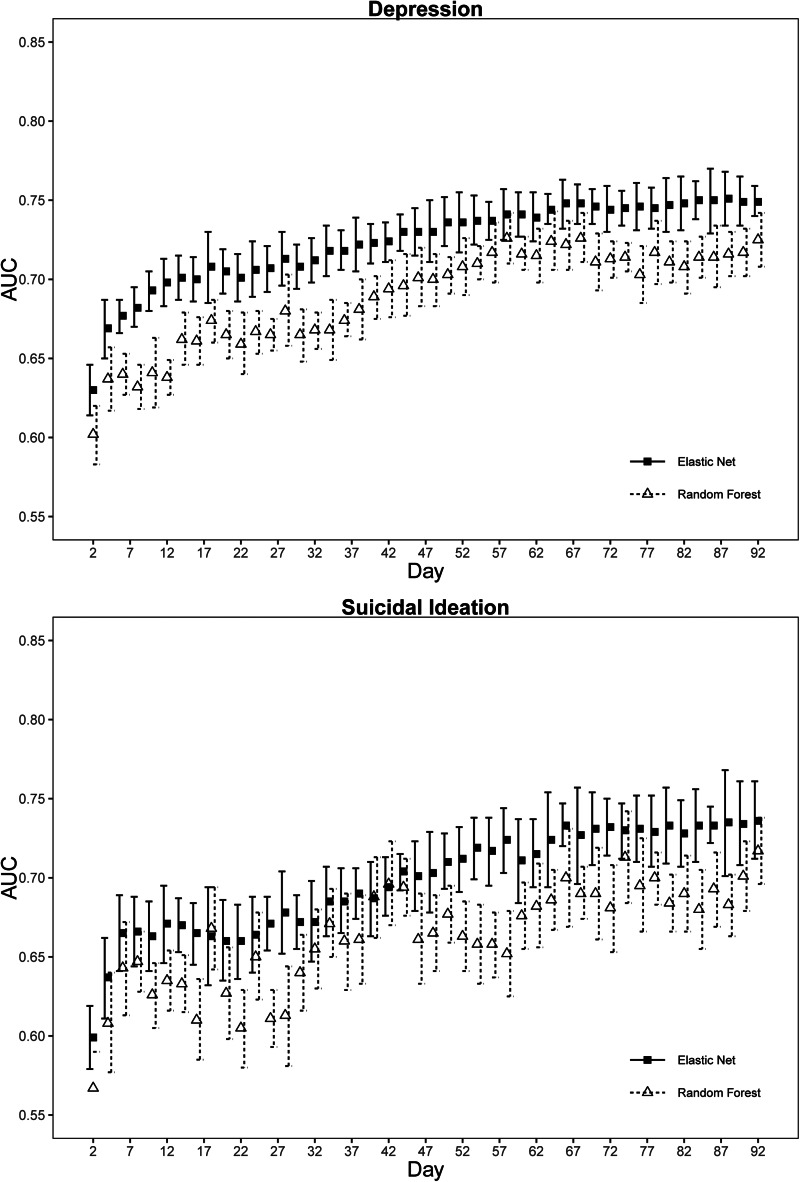
Full sample ENR and RF performance (mood predictors only).

As shown in Table 1, the AUC for predicting the presence of depression using the full 92 days of data was best when using the ENR, with similar results when using all variables (AUC = 0.750) or mood-only variables (AUC = 0.749). Prediction accuracy was within 1 standard error of the full-model AUC by weeks 7–8 of the quarter and maintained acceptable accuracy (AUC > 0.70) after only 14 days of data collection. These ENR models attained better overall AUCs (0.749–0.750) relative to the non-linear RF models (0.704–0.725; see Fig. 1).

**Table 1.** Simple and complex ML models

	Day 92 mean AUC (s.e.)	Day 92 Sens/Spec	Day *n*^a^	Day *n* mean AUC	Day *x*^b^
Depression (ENR)					
Mood variables	0.749 (0.010)	0.721/0.686	57	0.741	14
All variables	0.750 (0.017)	0.726/0.692	48	0.735	15
Depression (RF)					
Mood variables	0.725 (0.017)	0.721/0.669	51	0.708	48
All variables	0.704 (0.012)	0.675/0.692	48	0.693	90
SI (ENR)					
Mood variables	0.736 (0.025)	0.701/0.727	50	0.717	42
All variables	0.699 (0.028)	0.671/0.694	42	0.672	N/A
SI RF					
Mood variables	0.717 (0.021)	0.713/0.699	41	0.696	90
All variables	0.663 (0.016)	0.642/0.663	81	0.656	N/A

AUC, area under the curve; ENR, elastic net regression; RF, random forest; SI, suicidal ideation; N/A, did not meet specified threshold during study period.

a First day with mean AUC within 1 standard error of day 92 mean AUC.

b Last day with mean AUC < 0.70.

For SI, the ENR model using only mood variables over 92 days provided better predictive accuracy for SI (AUC = 0.736) relative to the model incorporating passive sensing (AUC = 0.699). Prediction accuracy was within 1 standard error of the full-model AUC at a similar timeframe (weeks 7–8) as depression.^†^^1^ Acceptable accuracy (AUC > 0.70) in the mood-only ENR model was maintained by week 7 but did not consistently meet this threshold when sensor data were included. As with depression, the ENR models attained better overall AUCs (0.699–0.736) relative to the non-linear RF models (0.663–0.717; see Fig. 1).

### Influence of missingness

**Fig. 2.**
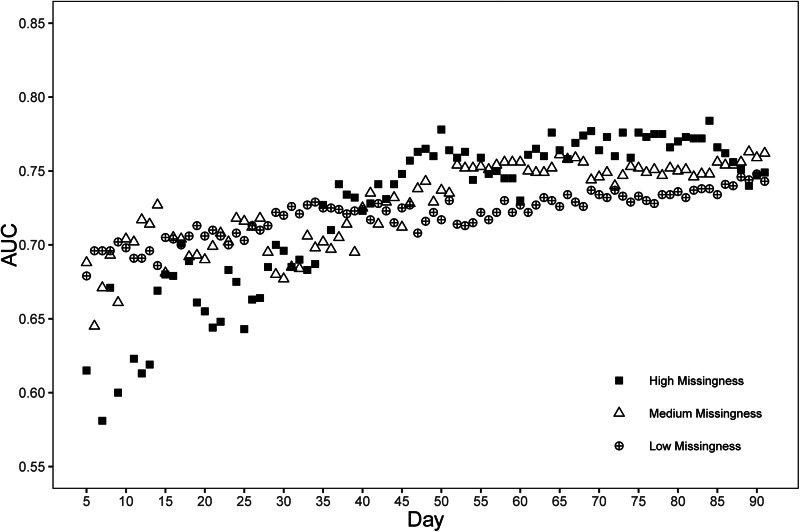
Depression model performance by rates of missingness (ENR with mood predictors only).

Rates of missing daily mood ratings (using best model configuration from the full sample – ENR with mood-only) impacted predictions of depression across the study period. Model performance for participants with different rates of missingness is presented in Table 2 and Fig. 2. Participants with low missingness (response rate >67%) demonstrated acceptable accuracy after only 2 weeks and were within 1 standard error of the final model accuracy by the end of the first month. Notably, participants with medium degrees of missingness attained a higher overall level of accuracy in the full and final model (AUC = 0.762), but did not maintain acceptable accuracy until after 6 weeks of data collection. Participants with high missingness (response rate <33%) were difficult to predict during the first several weeks, but by week 5 maintained acceptable accuracy (similar to the medium missingness group).

**Table 2.** Missingness and prediction accuracy

	Day 92 mean AUC (s.e.)	Day 92 Sens/Spec	Day *n*^a^	Day *n* mean AUC	Day *x*^b^
Depression					
Low	0.743 (0.024)	0.725/0.687	29	0.722	14
Medium	0.762 (0.018)	0.815/0.657	52	0.752	39
High	0.749 (0.023)	0.698/0.758	35	0.727	34
SI					
Low	0.780 (0.021)	0.825/0.700	73	0.764	44
High	0.702 (0.037)	0.686/0.667	6	0.711	90

AUC, area under the curve; SI, suicidal ideation.

Models were generated using the ENR models containing only mood variables. For depression, missingness rates were calculated based on completion of daily mood surveys at the following levels: low >66%, medium 34–65%, high <33%. Due to lower incidence of SI, missingness was constrained to above (low) or below (high) completion rates of 50%.

a First day with mean AUC within 1 standard error of day 92 mean AUC.

b Last day with mean AUC < 0.70.

**Fig. 3.**
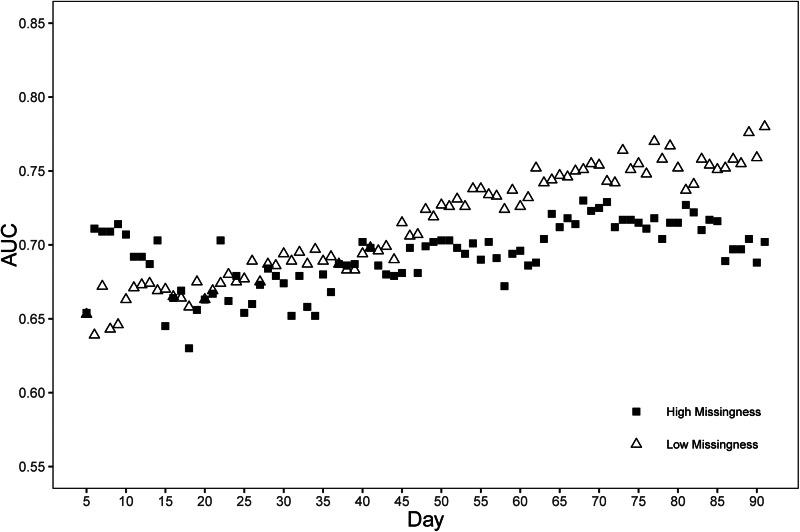
SI model performance by rates of missingness (ENR with mood predictors only).

Rates of missing daily mood ratings (using best model configuration from the full sample – ENR with mood-only) also impacted predictions of SI (see Table 2; Fig. 3). For those with low missingness (response rate >50%), prediction accuracy steadily improved throughout the quarter, attaining a final AUC of 0.780, but required 6–7 weeks to maintain acceptable accuracy. The high missingness group (response rate <50%) was within the standard error of the final model after only 1 week, but in contrast to the low missingness group, did not improve much during the quarter and was not consistently above the acceptable accuracy threshold (AUC < 0.70).

## Discussion

This is the first study, to our knowledge, to utilize ILMs to predict depression and SI in a non-clinical sample across different time scales. The study's primary objective was to investigate relative benefits and tradeoffs concerning shorter *v.* more extended (until depression and ideation were assessed 92 days later) intensive data collection protocols. Our findings suggest that simple, daily mood assessments can predict depression 3 months out with reasonable accuracy after only 2 weeks, and that improvement in prediction accuracy for these distal outcomes tend to level off after about 7 weeks. Conversely, more time and assessment may be needed to predict SI accurately (approximately 7–8 weeks of data). Moreover, these general patterns varied as a function of adherence. As it pertains to missing data and depression, those responding more consistently had accurate predictions sooner, while those with high rates of missingness achieved similar predictive accuracy after approximately 5 weeks. With respect to missingness and SI, predictive accuracy was quite weak among those who responded inconsistently, even as assessment continued over time, whereas those with lower rates of missingness had steady improvements in predictive accuracy into the latter weeks of the 3 month period. Overall, the study's results suggest that, given the high participant burden and low adherence associated with ILMs over long periods of time, there may be limited value in continued data collection after about 7 weeks in this naturalistic sample. A key exception was observed for predicting SI, which continued to improve in accuracy with additional data collection. There may be key differences in the stability of depression and SI that places greater value on more proximate assessments for the prediction of SI, at least with respect to daily mood assessments.

Another notable finding in this study was that, despite enthusiasm for the role of passive sensing and complex ML methods in mental health research (Burke et al., 2019; Colombo et al., 2019), simpler algorithms (ENRs) with only mood-derived predictors were either equivalent or outperformed more complex RF models with mood and passive-sensing (Fitbit) predictors. This is likely due to the implicit feature selection performed by the elastic net algorithm shrinking the weights of the Fitbit variables, and the fact that inclusion of Fitbit predictors in the more complex RF non-linear algorithms did not improve performance over mood data alone. The sharp decrease in performance for RF models when Fitbit variables were included suggests these variables were simply adding noise, rather than a meaningful predictive signal. While there remains significant potential, and a reduction of burden, associated with passive sensing, these features require additional refinement to maximize their potential (Sheikh, Qassem, & Kyriacou, 2021).

In the current study, end-of-quarter depression was detected with acceptable accuracy as early as the first 2 weeks of internship. Given this potential to detect early states of vulnerability, particularly depression, under naturalistic conditions, there are significant implications for preventative interventions. Specifically, adaptive interventions use ongoing information about individuals' functioning or treatment progress to modify aspects pertaining to the type, intensity, or modality used to deliver the treatment (Nahum-Shani et al., 2012). Examining early signs of impending depressive symptoms or SI could be beneficial for operationalizing when and for whom support is indicated, and as soon as such signs are identified. More work is needed to further examine the utility of ILMs in detecting states of vulnerability to mental health outcomes to guide personalized interventions by specifying the conditions in which interventions should be provided, in different contexts and populations.

Our work also highlights tradeoffs concerning maximizing predictive accuracy and minimizing response burden, and when such balance can be achieved (e.g. low *v.* high missingness). Briefer ILD protocols that achieve similar predictive accuracy may be more practical in naturalistic studies or clinical contexts. For example, model performance using daily diary data in the weeks following psychiatric hospitalization was similar when using 1 *v.* 2 weeks of data in predicting subsequent suicidal crises (Czyz, Koo, Al-Dajani, King, & Nahum-Shani, 2021). Given the challenges associated with participation decay over time in intensive longitudinal studies (e.g. Czyz et al., 2018; Glenn et al., 2020), our findings suggest there may be opportunities to modify assessment schedules to lessen attrition without compromising predictive validity.

## Limitations

While this study has many strengths, including a very large sample incorporating self-report and passive-sensing ILD, as well as a rigorous statistical approach, our findings must be taken within the context of study limitations. With respect to measurement, while the PHQ-9 is widely used as a screener and tracker of depressive symptoms, scores do not necessarily reflect a clinical diagnosis of depression. Likewise, our measure of SI from this scale does not permit the distinction between passive and active suicidal thoughts, including the presence of its more severe forms (e.g. ideation with methods or intent). Our sample of medical interns was fairly diverse with respect to gender and race/ethnicity, yet the medical intern population is inherently homogenous with respect to education and occupation. There may also be aspects of the intern work schedule that result in patterns of sleep or activity that do not mirror the general population with respect to associations with depression and SI, resulting in non-significant findings for passive-sensing data. Intensive longitudinal designs often examine both proximal (i.e. next-day) and distal outcomes (i.e. months), and it should be noted that our findings are primarily focused on distal outcomes – we would not expect our predictive accuracy findings over time to follow the same patterns for more proximal outcomes. We note that various hierarchical structures may be present for these data (e.g. individuals nested within institutions or within specialties). We had no specific hypotheses about differences across these higher-level factors and we opted not to specify nesting within our models. However, modeling such dependencies could potentially improve predictive performance in future studies. We also acknowledge that we only compared two types of ML models, and that results may differ across other ML models of varying complexity. Finally, it is worth noting that the overall model accuracy for predicting depression and SI was merely good, but not excellent (AUC < 0.80), and there is room to improve methods for both passive-sensing and self-report assessments.

## Conclusions

ILMs provide significant opportunities for improved monitoring of mental health conditions and risk detection for both proximal and distal mental health outcomes. Despite enthusiasm for sophisticated ML methods, simpler ENRs based only on self-reported data outperformed the more complex RF models incorporating both self-report and passing-sensing features. While data were collected daily for 13 weeks, predictive accuracy for depression and SI improved minimally after weeks 7–8, suggesting that daily data collection for more than 2 months may have limited value for predicting distal outcomes. Additionally, acceptable predictive accuracy for moderate-to-severe depression at the end of the quarter was attained after only 2 weeks, highlighting the potential and need for early interventions that can adaptively respond to individuals at risk for these negative mental health outcomes.

## Footnotes

† The notes appear after the main text.

## Note

1 To test the stability of these findings, mood-only models using ENR also examined days to accuracy within 1 standard error of the 92 day model for more distal depression and suicidal ideation (end of second quarter). Results were similar to the first quarter (day 58 for depression, day 52 for ideation), albeit with lower overall accuracy (full model AUC = 0.70 for depression, AUC = 0.67 for suicidal ideation).
